# Editorial: Bioactive containing plant based waste for (nano)-biocomposites: applications in biomedicine, health, and bioremediation

**DOI:** 10.3389/fchem.2025.1575200

**Published:** 2025-02-28

**Authors:** Ram Prasad, Juan Bueno

**Affiliations:** ^1^ Department of Botany, Mahatma Gandhi Central University, Motihari, Bihar, India; ^2^ Fundación Centro de Investigación y Biotecnología de la Biodiversidad (BIOLABB), Armenia, Colombia

**Keywords:** plant based waste, bioactivity, (nano)-biocomposites, circular economy, waste management

Biotechnological waste includes waste from laboratories, clinical settings, and other sources. It can encompass biological, chemical, and organic waste components (Brandão et al., 2021). Thus, waste that can be transformed through biotechnological processes incorporates biological and plastic waste (Chavan et al., 2022). This type of organic waste is also based on plants and is produced by the food and beverage industry, the paper industry, and agriculture. Based on this, plant-based waste is defined as any waste that comes from plants, such as food waste, fallen leaves, and agricultural byproducts (Salazar Sandoval et al., 2024). For this reason, the biotechnological processing of plant-based organic waste can serve as a source for the production of new bioactive compounds that can be enhanced by nanotechnology to increase their stability and bioactivity and reduce their toxicity (Leta et al., 2024). This, Research Topic was proposed: Bioactive plant-based waste for (nano)-biocomposites: Applications in biomedicine, health, and bioremediation. Its aim is to explore nanobiotechnological solutions and innovations in the optimization of the resources obtained from the processing of plants, to develop new products to advance humankind’s quality of life.

Among the studies in this special issue, in the one submitted by Bariş and Kötekoğlu the authors developed an organogelator, a molecule that, when dissolved in an organic solvent, forms a gel. It can be used in small quantities and has applications in cosmetics, optics, and other fields (Zeng et al., 2021). The compound developed in this research was 1(N1,N2-bis(2S,3S)-1-((4-(tert-butyl)benzyl)amino)-3-methyl-1-oxopentan-2yl)oxalamide), which is biocompatible with high penetration capacity, and is useful as a transporter of topical bioactive molecules with great stability. Also, to determine the capacity to capture the gels obtained, non-steroidal anti-inflammatory drugs (NSAIDs) ibuprofen and naproxen were used, which are safer for muscle pain in topical applications, due to that gastric intolerance that both drugs can produce (Manoukian et al., 2017). Ultimately, this research demonstrates the ability of novel organogels as a carrier system for drugs and bioactive molecules for medical use. Thus, organogels and oleogels are promising formulations for the delivery of useful bioactive compounds from agricultural and food waste (Souza and Pereira, 2023).

Ullah et al. proposed essential oils as the basis of new biocompositions that can use the waste of medicinal plants with biological activity as an extraction source (Mohamed Abdoul-Latif et al., 2023). In this research, the authors evaluated essential oils (cardamom, cinnamon, and clove) by their ability to inhibit the conformation of the biofilms formed by the *Staphylococcus aureus* and *Staphylococcus epidermidis*; They also used the *Chromobacterium violaceum* model to determine the inhibition of the phenomenon of quorum sensing which is necessary to group the microbial cells in the exopolysaccharide matrix of the biofilm (Dimitrova et al., 2023). Finally, the druggability of the majority of the essential oil compounds (Cinnamaldehyde, Eugenol, and Cineole) was determined in combination with Carbopol 940, a biopolymer used as a gelling agent. It is important to develop oral hygiene products with bioactive components from essential oils in Carbopol 940 that allow for the management of biofilms, caries, and gingivitis (Chelu, 2024).

Mashal et al. evaluated polyphenolic compounds (ferulic acid, gentisic acid, gallic acid, rutin, quercetin, catechin, caffeic acid, syringic acid, and vanillic acid) against *Escherichia coli* (ATCC 25922), *Klebsiella pneumoniae* (ATCC BAA-1705), *Pseudomonas aeruginosa* (ATCC 15442), and *S. aureus* (ATCC 33862). These same compounds were evaluated against isolated biofilms from dental plaque from female diabetic patients with the following microbial population: *Bacillus chungangensis*-1, *P. aeruginosa*, *Bacillus paramycoides*, *Bacillus chungangensis*-2, and *Paenibacillus dendritiformis*. As a result, the polyphenolic compounds showed antimicrobial, antibiofilm, and antiquorum-sensing activity in the *in vitro* models. Druggability was determined by looking for compliance with Lipinski’s rule of five, a guideline that predicts whether a chemical compound can be an orally active drug in humans. The rule is based on four guidelines, each with a number that is a multiple of five (Karami et al., 2022): molecular weight: less than 500 Da (MW < 500 Da), hydrogen bond donors: less than 5 (H−bond donors < 5), hydrogen bond acceptors: less than 10 (H−bond acceptors < 10), Calculated Log P: less than 5 (CLogP < 5). Finally, the ADMET profile was tested, showing that rutin, quercetin, and catechin have high oral absorption, while gallic acid and rutin present low intestinal absorption. This study is of great importance in determining how bioactive natural products can be used in pharmacologically stable formulations for medical applications (Dzobo, 2022).

Doğan et al. developed a magnetic nanocomposite functionalized with flavonol morin, which is an anticarcinogenic compound (Mottaghi and Abbaszadeh, 2021). This functionalized nanostructure was synthesized using the fruit of *Celtis tournefortii* (endemic tree of the southwestern slopes of Mt. Etna and the Nebrodi mountain range) to obtain a composite with activated carbon named CtAC. Later CtAC was employed for the synthesis of the IONPs@CtAC magnetic nanocomposite and loaded with morin; it was evaluated for cytotoxicity against HT-29 (colorectal), T98-G (glioblastoma) cancer cell lines, and human umbilical vein endothelial cell (HUVEC) as a healthy cell line. The authors demonstrated that this nanocomposite increased morin release, solubility and dissolution. Also, it was found to be active against tumor cell lines, which makes it an interesting nano-delivery system. These results are important for the development of nanodrugs functionalized with natural compounds for cancer treatment because natural compounds encapsulated in nanoparticles can be used to treat cancer more effectively and safely. This is because nanoparticles can improve the bioavailability and pharmacokinetics of natural compounds, while also reducing their adverse effects on healthy cells (Zhao et al., 2020).

In this order of ideas, It is possible to join three fields of applied science to develop a global proposal of circular bio-economy, namely: biotechnology, nanotechnology, and synthetic biology. With this integration model, it is possible to have a great impact on medicine, food, agriculture, and environmental sustainability (Venkatesh, 2022; Osherov et al., 2023).
